# Assessment of Differentiated Thyroid Carcinomas in French Polynesia After Atmospheric Nuclear Tests Performed by France

**DOI:** 10.1001/jamanetworkopen.2023.11908

**Published:** 2023-05-05

**Authors:** Florent de Vathaire, Monia Zidane, Constance Xhaard, Vincent Souchard, Sylvie Chevillard, Catherine Ory, Frédérique Rachédi, Sébastien Nunez, Axelle Leufroy, Laurent Noël, Thierry Guérin, Larys Shan, Frédérique Bost-Bezeaud, Patrice Petitdier, Gilles Soubiran, Rodrigue Allodji, Yan Ren, Françoise Doyon, Marc Taquet, Jacques Gardon, André Bouville, Vladimir Drozdovitch

**Affiliations:** 1 Radiation Epidemiology Team, Centre de Recherche en Epidémiologie en Santé des Populations, Institut National de la Santé et de la Recherche Médicale, Unit 1018, Villejuif, France; 2 Gustave Roussy, Villejuif, France; 3 University Paris Saclay, Villejuif, France; 4 Now with University of Lorraine, Institut National de la Santé et de la Recherche Médicale, CIC 1433, Nancy, Centre HospitalierRegional Universitaire, U1116, Nancy, France; 5 Laboratoire de recherche sur la Réparation et la Transcription dans les Cellules Souches Hématopoïétiques, Institut de Biologie François Jacob, Institut de Recherche en Cancérologie de Montpellier, Direction de la Recherche Fondamentale, Commissariat à l'Énergie Atomique, 92265 Fontenay-aux-Roses Cedex, France; 6 University Paris-Saclay, 92265 Fontenay-aux-Roses Cedex, France; 7 Endocrinology Unit, Centre Hospitalier Territorial, Tahiti, French Polynesia; 8 Agence Nationale Sécurité Sanitaire Alimentaire Nationale, Laboratory for Food Safety, F94700 Maisons-Alfort, France; 9 French Directorate General for Food, Ministry of Agriculture, Agro-16 Food and Forestry, Paris, France; 10 Agence Nationale Sécurité Sanitaire Alimentaire Nationale, Strategy and Programmes Department, Maisons-Alfort, France; 11 Private practice, Tahiti, French Polynesia; 12 Laboratory of Anatomy and Cytopathology, Centre Hospitalier Territorial, Tahiti, French Polynesia; 13 Research Institute for Development, Arue, Tahiti, French Polynesia; 14 HydroSciences Montpellier, Univ Montpellier, Research Institute for Development, Centre National de la Recherche Scientifique, Montpellier, France; 15 Division of Cancer Epidemiology and Genetics, National Cancer Institute, National Institutes of Health, Department of Health and Human Services, Bethesda, Maryland

## Abstract

**Importance:**

Due to the amount of iodine 131 released in nuclear tests and its active uptake by the thyroid, differentiated thyroid carcinoma (DTC) is the most serious health risk for the population living near sites of nuclear tests. Whether low doses to the thyroid from nuclear fallout are associated with increased risk of thyroid cancer remains a controversial issue in medicine and public health, and a misunderstanding of this issue may be associated with overdiagnosis of DTCs.

**Design, Setting, and Participants:**

This case-control study was conducted by extending a case-control study published in 2010 that included DTCs diagnosed between 1984 and 2003 by adding DTCs diagnosed between 2004 and 2016 and improving the dose assessment methodology. Data on 41 atmospheric nuclear tests conducted by France between 1966 and 1974 in French Polynesia (FP) were assessed from original internal radiation-protection reports, which the French military declassified in 2013 and which included measurements in soil, air, water, milk, and food in all FP archipelagos. These original reports led to an upward reassessment of the nuclear fallout from the tests and a doubling of estimates of the mean thyroid radiation dose received by inhabitants from 2 mGy to nearly 5 mGy. Included patients were diagnosed from 1984 to 2016 with DTC at age 55 years or younger and were born in and resided in FP at diagnosis; 395 of 457 eligible cases were included, and up to 2 controls per case nearest by birthdate and matched on sex were identified from the FP birth registry. Data were analyzed from March 2019 through October 2021.

**Exposure:**

The radiation dose to the thyroid gland was estimated using recently declassified original radiation-protection service reports, meteorological reports, self-reported lifestyle information, and group interviews of key informants and female individuals who had children at the time of these tests.

**Main Outcomes and Measures:**

The lifetime risk of DTC based on Biological Effects of Ionizing Radiation (BEIR) VII models was estimated.

**Results:**

A total of 395 DTC cases (336 females [85.1%]; mean [SD] age at end of follow-up, 43.6 [12.9] years) and 555 controls (473 females [85.2%]; mean [SD] age at end of follow-up, 42.3 [12.5] years) were included. No association was found between thyroid radiation dose received before age 15 years and risk of DTC (excess relative risk [ERR] per milligray, 0.04; 95% CI, −0.09 to 0.17; *P* = .27). When excluding unifocal noninvasive microcarcinomas, the dose response was significant (ERR per milligray, 0.09; 95% CI, −0.03 to 0.02; *P* = .02), but several incoherencies with the results of the initial study reduce the credibility of this result. The lifetime risk for the entire FP population was 29 cases of DTC (95% CI, 8-97 cases), or 2.3% (95% CI, 0.6%-7.7%) of 1524 sporadic DTC cases in this population.

**Conclusions and Relevance:**

This case-control study found that French nuclear tests were associated with an increase in lifetime risk of PTC in FP residents of 29 cases of PTC. This finding suggests that the number of thyroid cancer cases and the true order of magnitude of health outcomes associated with these nuclear tests were small, which may reassure populations of this Pacific territory.

## Key Points

**Question:**

Were 41 atmospheric nuclear tests carried out by France in French Polynesia between 1966 and 1974, whose nuclear fallout levels were reassessed upward, associated with increased thyroid cancer risk in French Polynesia residents?

**Findings:**

This case-control study including 395 cases of differentiated thyroid carcinomas (DTCs) diagnosed between 1983 and 2016 and 555 controls, there was no clear association between thyroid radiation dose received and the risk of DTCs. In models, French nuclear testing was associated with 2.3% of lifetime risk of DTCs.

**Meaning:**

This study, using data from well-documented nuclear testing in declassified original reports, may suggest the true order of magnitude of the health outcomes associated with French nuclear testing in French Polynesia.

## Teaser

This case-control study examines the association of radiation exposure from French atmospheric nuclear tests and risk of thyroid cancer among French Polynesia residents.

## Introduction

Between 1966 and 1974, France conducted 41 atmospheric nuclear weapon tests in Mururoa and Fangataufa in the southeastern part of the Tuamotu-Gambier archipelago of French Polynesia (FP).^1^ To evaluate the potential association of fallout from atmospheric nuclear tests with the incidence of differentiated thyroid carcinoma (DTC) observed in FP, we performed a case-control study between 2003 and 2006 including 229 DTC cases diagnosed before 2003 individually matched on sex and birth date to 373 controls from the general population. This study permitted us to better understand risk factors associated with DTC in this population,^2,3,4,5^ in particular the association of a large number of pregnancies^2^ and body mass index with a large increase in risk.^3^ We also showed that a diet closer to the traditional Polynesian diet could be associated with protection from DTC^6,7^ and identified FP as an area of moderate iodine deficiency, where an increased diary iodine intake may be associated with a lower risk of thyroid cancer.^7^

We published in 2008 a first estimation of ionizing radiation doses to the thyroid received from nuclear tests; this estimate was based on available meteorological data, data from individual dietary questionnaires, and data from scientific reports sent by France to the United Nations Scientific Committee on the Effects of Atomic Radiation (UNSCEAR) after each series of tests.^8^ The mean estimated thyroid dose from exposure to radioactive fallout was very low, at 2.6 mGy for cases and controls, which received mean doses of radiation of 1.8 mGy and 1.7 mGy, respectively, before age 15 years. Although this study was based on small numbers, an association between the radiation dose to the thyroid before age 15 years and risk of thyroid cancer was found.^9^

In 2013, France declassified the original internal reports of the radioprotection services in charge of radiation protection during the entire period of nuclear tests conducted in FP. Based on these reports, it was concluded in some publications that health outcomes associated with French nuclear tests may have been more substantial than previously estimated.^10,11^ We took the opportunity of this declassification to extend the DTC case-control study by adding DTC cases diagnosed from 2004 to 2016 and improving its dosimetric reconstruction.

## Methods

This case-control study was approved by the French Commission Nationale de l’Informatique et des Libertés and the Ethical Committee of French Polynesia, and each participant signed an individual written consent form. The reporting of this study follows the Strengthening the Reporting of Observational Studies in Epidemiology (STROBE) reporting guideline for the reporting of case-control studies.

### Study Population

The population and methodology of phase 1 of the case-control study have already been described.^1,9^ Patients diagnosed between 1984 and 2003 with DTC at age 55 years or younger who were born in FP and resided in FP at the time of diagnosis were eligible. Of 255 eligible DTC cases, 229 were included. For each case, 2 controls nearest by date of birth and matched on sex were identified from the FP birth registry. Among 458 eligible controls, 373 were interviewed. Cases and controls were interviewed face to face, with questions on ethnic group, education, occupation, place of residence, weight history, personal and familial history of thyroid disease and cancer, gynecologic and reproductive history, and medical chest radiograph exposure and diet at the time of the interview and during childhood. Ethnic group was self-reported by participants in response to the question “How do you define the ethnic group of each of your parents?” Available categories were Asian, European, Polynesian, Polynesian Asian, Polynesian European, and other (≥1 parent with self-defined ethnicity of a group other than Polynesian, Asian, or European). Ethnic groups were analyzed because of a possible difference in radiation sensibility of the thyroid among people of Polynesian ethnicity compared with other ethnic groups. Iodine content in traditional FP food was estimated by analyzing vegetable, fruit, seafood, and lagoon and ocean fishes from all Polynesian archipelagos using a methodology permitting reduction of iodine evaporation during sample preparation.^12^

Phase 2 of the case-control study was carried out between 2015 and 2018 in the same way and using the same questionnaire and interview methodology as in phase 1; however, the criteria of selection were relaxed for birth date, and the Cancer Registry of FP and the Caisse de Prévoyance Sociale of FP did not participate in the study. A total of 202 potential cases not included in phase 1 were identified from medical files of hospitals and private endocrinologists in FP. The latest addresses of potential cases were obtained from medical files. Of these potential cases, 7 individuals died at the time of interview, 11 were not found, and 18 refused to participate. The remaining 166 patients were included, among whom 156 individuals were diagnosed between 2004 and 2016 and 10 were diagnosed between 1995 and 2003; these 10 patients were not included in the initial study because they could not be found when the study was conducted. For each potential case, the Institut de la Statistique de Polynésie Française provided the list of all people born the same day, extracted from the civil status file. We randomly selected controls from this list, and we obtained last addresses from phone directories, medical doctors, and local government records. In case of death, impossibility to trace, or refusal to participate, the potential control was replaced. At the end of the study, 17 potential controls had refused and 182 had been interviewed. Lastly, some controls were individually rematched with the case with the closest date of birth to avoid singletons.

Over 2 phases, the study included a total of 395 of 457 eligible cases diagnosed from 1984 to 2016. The overall flowchart of the study is reported in the eFigure in Supplement 1. Main characteristics of cases included in phase 1 and 2 of the study are reported in eTable 1 in Supplement 1.

### Estimation of Thyroid Radiation Dose

Methodology and results of the dose estimation process have already been described.^13,14,15^ We reconstructed thyroid radiation doses for each study participant due to 3 sources: (1) intake of iodine 131 (^131^I), short-lived radioiodine isotopes (^132^I, ^133^I, and ^135^I), and tellurium 132 (^132^Te) via inhalation and ingestion of food and drinking water; (2) external irradiation from γ-emitting radionuclides deposited on the ground; and (3) ingestion of long-lived cesium (^137^Cs) with food.

The dosimetry model used in phase 1 of the study^8^ was substantially improved. Results of radiation monitoring of the environment and food performed by the Service Mixte de Sécurité Radiologique and Service Mixte de Contrôle Biologique, the internal radiation-protection services of the French Army and French Alternative Energies and Atomic Energy Commission, after each test were used. Results, which were declassified in 2013, were used instead of syntheses sent by France to UNSCEAR, which were used in the initial study. These reports made it possible to conduct a comprehensive estimation of the ground deposition of 33 radionuclides at the time of arrival of fallout after each test based on measurements of total ground deposition, total β concentration in the air, or exposure rates at different locations in FP.^13^ In 2016 and 2017, we collected historical data by questionnaire on population lifestyle related to the period of the tests; these data were integrated using focus group discussions and key informant interviews monitored in each of the 5 archipelagos of FP. Additionally, declarations reported in self-questionnaires were used.^14^

This new dosimetric reconstruction was used to estimate the mean lifetime thyroid dose among study participants at approximately 4.7 mGy.^15^ This value was approximately 2 times higher than the 2.7 mGy^8^ estimated in the first dosimetric reconstruction, but the highest dose in the new reconstruction was estimated to be approximately 36 mGy,^15^ a value similar to the maximum dose of 37 mGy estimated in the first reconstruction.^8^ Doses of ^131^I intake ranged up to 27 mGy, while those from intake of short-lived iodine isotopes (^132^I, ^133^I, and ^135^I) and ^132^Te ranged up to 14 mGy. Thyroid doses from external exposure ranged up to 6 mGy, and those from internal exposure due to ^137^Cs ingestion did not exceed 1 mGy. Intake of ^131^I was the main pathway of thyroid exposure, accounting for 72% of the total dose.^15^

### Statistical Analysis

Data were analyzed using conditional logistic regression with the Epicure epidemiological software version 2 (Hirosoft International Corp)^16^ and SAS statistical software version 9.4 (SAS Institute). To investigate the association between radiation dose to the thyroid and the risk of DTC, we compared nested models using likelihood-ratio tests.^17^ Tests for linear trend were also performed. Data were analyzed from March 2019 through October 2021.

The analysis of the association of radiation with the risk of thyroid cancer was systematically adjusted for ethnic group of the parents, level of education, height, body mass index score, familial history of thyroid benign pathology, personal history of radiation therapy, familial history of cancer, number of pregnancies, and dietary iodine intake at the time of interview. Analyses were also adjusted by interviewer. The ethnic group of the parents was analyzed as both Polynesian vs others to investigate a possible specific radiosensibility among people of the Polynesian ethnic group and because of a small number of cases in our study. Only 2-sided tests were used in the analysis, with the level of significance set at *P* = .05.

Thyroid microcarcinoma discovery depends on clinical investigations, which may have been more intense in the population known to have been exposed. A study^18^ found that in 2015, 70% of FP medical doctors believed that French nuclear tests induced thyroid cancer in FP residents, an opinion that very probably was associated with overscreening. A first sensitivity analysis was therefore performed in our study, excluding cases with unifocal tumors of 10 mm or less without extrathyroidal invasion and their controls. There was a loss in power owing to the high number of controls excluded from this first sensitivity analysis, and bias could have occurred in this conditional logistic regression analysis if no exact matching was done.^19^ Therefore, we also performed a second sensitivity analysis excluding cases with unifocal tumors of 10 mm or less without extrathyroidal invasion but not their controls; we used unconditional logistic regression stratified on sex and 5-year age classes (ages 10-19, 15-19, 20-24, 25-29, 30-34, 35-39, 40-44, 45-49, 50-54, 55-59, 60-64, and 65-83 years).

We also estimated lifetime thyroid cancer risk in FP associated with fallouts from nuclear atmospheric tests based on the model defined for thyroid cancer in the 2006 report of the National Academies of Sciences’ Biological Effects of Ionizing Radiation (BEIR) VII Committee.^20^ In this model, the estimated relative risk (ERR) per gray does not depend on the time elapsed since irradiation or on the dose rate but only on sex and age at irradiation. The ERR per gray is 1.5 (95% CI, 0.28-3.90) for women and 0.53 (95% CI, 0.14-2.0) for men aged 30 years or older at the time of irradiation; these values are multiplied by exp(−0.83 × (age at irradiation − 30)/10) if the age is less than 30 years at irradiation.^20^ For this estimation, we used sex- and age-specific thyroid cancer incidence rates published in volumes VII^21^ for calendar years before 1995 and IX for 1995 and afterward^22^ of *Cancer Incidence in Five Continents*, which provide the only cancer incidence data available for FP; these sources reported, respectively, a total of 67 and 216 incident cases. More recent data were published online^23^ and were not detailed by age, according to which a total of 43 incident thyroid cancers were diagnosed in FP in 2016, a lower incidence than in 1998 to 2002.

To simplify calculations, lifetime thyroid cancer risk was estimated under assumptions that (1) all atmospheric nuclear tests took place in 1971, (2) the thyroid radiation dose received by controls in our case-control studies who were alive and present in FP during the tests^15^ were representative of doses received by residents of the same archipelago in the same 5-year age group, and (3) mortality rates were the same in various archipelagos of FP since 1971. FP mortality rates during each calendar year, sex, and age were estimated from native populations registered in censuses of 1971, 1977, 1983, 1988, 1996, 2002, 2007, 2012, and 2017. These were then modeled for years afterward up to 2070, or until the extinction of the exposed population. These calendar-year–, sex-, and age-specific mortality rates were applied to anonymized individual data of the census of 1971 to estimate populations exposed to the risk of radiation-induced thyroid cancer from 1971 to 2070. The population distribution in each FP archipelago in the 1971 census is summarized in eTable 2 in Supplement 1, and eTable 3 in Supplement 1 shows the mean thyroid radiation dose by age group and main archipelago of residence.

We also used the online Radiation Risk Assessment Tool (RadRAT) version 4.3 (National Cancer Institute),^24^ which provides lifetime risks based on risk models for the 11 cancers included in the 2006 report of the National Academies of Sciences’ BEIR VII Committee.^20^ Because RadRAT does not include life tables or thyroid cancer reference incidence rates for the FP population, we used those for the Japanese population in 2010. This was done because among 2 Asian populations (Japanese and Korean) considered in this software, the Japanese population had a thyroid cancer incidence rate nearest to that among FP individuals.

## Results

A total of 395 cases of DTC (336 females [85.1%]; mean [SD] age at end of follow-up, 43.6 [12.9] years) and 555 controls (473 females [85.2%]; mean [SD] age at end of follow-up, 42.3 [12.5] years) matched on sex and age were included. Among cases, there were 219 individuals (55.6%) with 2 Polynesian parents; 57 individuals (14.5%) with Polynesian and Asian parents; 34 individuals (8.6%) with parents with Polynesian, Asian, and European origins; 71 individuals (18.0%) with Polynesian and European parents; and 14 individuals (3.5%) with parents with other ethnic origins. Among controls, there were 283 individuals (51.0%) with 2 Polynesian parents; 76 individuals (13.7%) with Polynesian and Asian parents; 47 individuals (8.5%) with parents with Polynesian, Asian, and European origins; 103 individuals (18.6%) with Polynesian and European parents; and 14 individuals (3.5%) with parents with other ethnic origins. Of the cases, 248 individuals (62.8%) were younger than age 15 years in 1966 or born during the tests (ie, potentially exposed during childhood), 94 individuals (23.8%) were aged 15 years or older in 1966, and 53 individuals (13.4%) were born in 1975 or later (ie, were never exposed). Of the 395 DTC cases, 137 cancers (34.7%) were unifocal microcarcinomas (Table 1; eTable 4 in Supplement 1).

**Table 1.** Characteristics of Thyroid Cancer Cases

Characteristic	Cases (N = 395), No. (%)
Sex	
Males	59 (14.9)
Females	336 (85.1)
Age at first test, y	
Born after tests	53 (13.4)
Born during tests	62 (15.7)
0-14	186 (47.1)
≥15	94 (23.8)
Age at diagnosis, y	
10-19	13 (3.3)
20-29	47 (11.9)
30-39	100 (25.3)
40-49	105 (26.6)
50-62	130 (32.9)
Histology	
Papillary	329 (79.2)
Follicular	66 (20.3)
Tumor size, mm	
Total with data, No.	346
≤10	171 (49.4))
11-40	137 (39.6)
>40	38 (11.0)
Unknown, No.	49
Multifocal tumor	
Total with data, No.	350
No	218 (62.3)
Yes	132 (37.7)
Unknown, No.	45
Bilateral tumor	
Total with data, No.	342
No	266 (77.8)
Yes	76 (22.2)
Unknown, No.	53

In a univariate conditional logistic regression, familial history of thyroid pathology (155 cases [39.2%] vs 96 controls [17.3%]; *P* < .001), familial history of thyroid cancer (20 cases [5.1%] vs 9 controls [1.6%]; *P* = .006), personal history of radiation therapy (34 cases [8.6%] vs 5 controls [0.9%]; *P* < .001), increased height, increased weight, increased BMI score, lack of a diploma, and insufficient dietary iodine intake were associated with increased risk of thyroid cancer. Among females, a higher number of pregnancies was associated with increased risk (40 cases [11.9%] vs 67 controls [14.2%] with 0 pregnancies; 162 cases [48.2%] vs 264 controls [55.8%] with 1-4 pregnancies; 134 cases [39.9%] vs 142 controls [30.0%] with 5-16 pregnancies; *P* = .002). The archipelago of birth was not associated with risk (Table 2; eTables 1 and 4 in Supplement 1).

**Table 2.** Characteristics of Cases vs Controls

Characteristic	Cases and controls, No. (%) (N = 950)		*P* value^a^
	Thyroid cancer cases (n = 395)	Controls (n = 555)	
Sex			
Males	59 (14.9)	82 (14.8)	NA^b^
Females	336 (85.1)	473 (85.2)	
Age at end of follow-up, mean (SD), y	43.6 (12.9)	42.3 (12.5)	NA^b^
Parent ethnic origin			
Both Polynesian	219 (55.4)	283 (51.0)	.09
Polynesian and Asian	57 (14.5)	76 (13.7)	
Polynesian, Asian, and European	34 (8.6)	47 (8.5)	
Polynesian and European	71 (18.0)	103 (18.6)	
Other^c^	14 (3.5)	76 (13.7)	
Familial history			
Thyroid cancer	20 (5.1)	9 (1.6)	.006
Thyroid pathology	155 (39.2)	96 (17.3)	<.001
Personal history of radiation therapy	34 (8.6)	5 (0.9)	<.001
Archipelago of birth			
Tahiti and Moorea	223 (56.5)	328 (59.1)	.81
Other Société islands	80 (20.2)	102 (18.4)	
Australes	25 (6.3)	30 (5.4)	
Marquises	18 (4.6)	18 (4.9)	
Tuamotu-Gambier	49 (12.4)	49 (12.3)	
Height, mean (SD), m			
Females	1.65 (0.06)	1.64 (0.06)	.001
Males	1.76 (0.07)	1.74 (0.07)	
Weight at end of follow-up, mean (SD), kg			
Females	84 (21)	75 (19)	<.001
Males	103 (23)	91 (19)	
BMI at end of follow-up, mean (SD)			
Females	30.7 (7.3)	27.9 (6.9)	<.001
Males	33.3 (6.9)	30.0 (5.9)	
Diploma			
No	161 (40.8)	197 (35.5)	.03
Yes	234 (59.2)	358 (64.5)	
Insufficient dietary intake			
No	282 (71.4)	427 (76.9)	.05
Yes	113 (28.6)	128 (23.1)	
Pregnancies, No.			
0	40 (11.9)	67 (14.2)	.002
1-4	162 (48.2)	264 (55.8)	
5-16	134 (39.9)	142 (30.0)	

Abbreviations: BMI, body mass index (calculated as weight in kilograms divided by height in meters squared); NA, not applicable.

^a^ Univariate conditional logistic regression.

^b^ *P* values are not available for these entries because there were matching criteria; by construction, the *P* value using a conditional logistic regression would be 1.0.

^c^ Other ethnicity indicates that at least 1 parent’s self-defined ethnicity was of a group other than Polynesian, Asian, or European.

### Radiation Dose to Thyroid Gland

**Figure 1.**
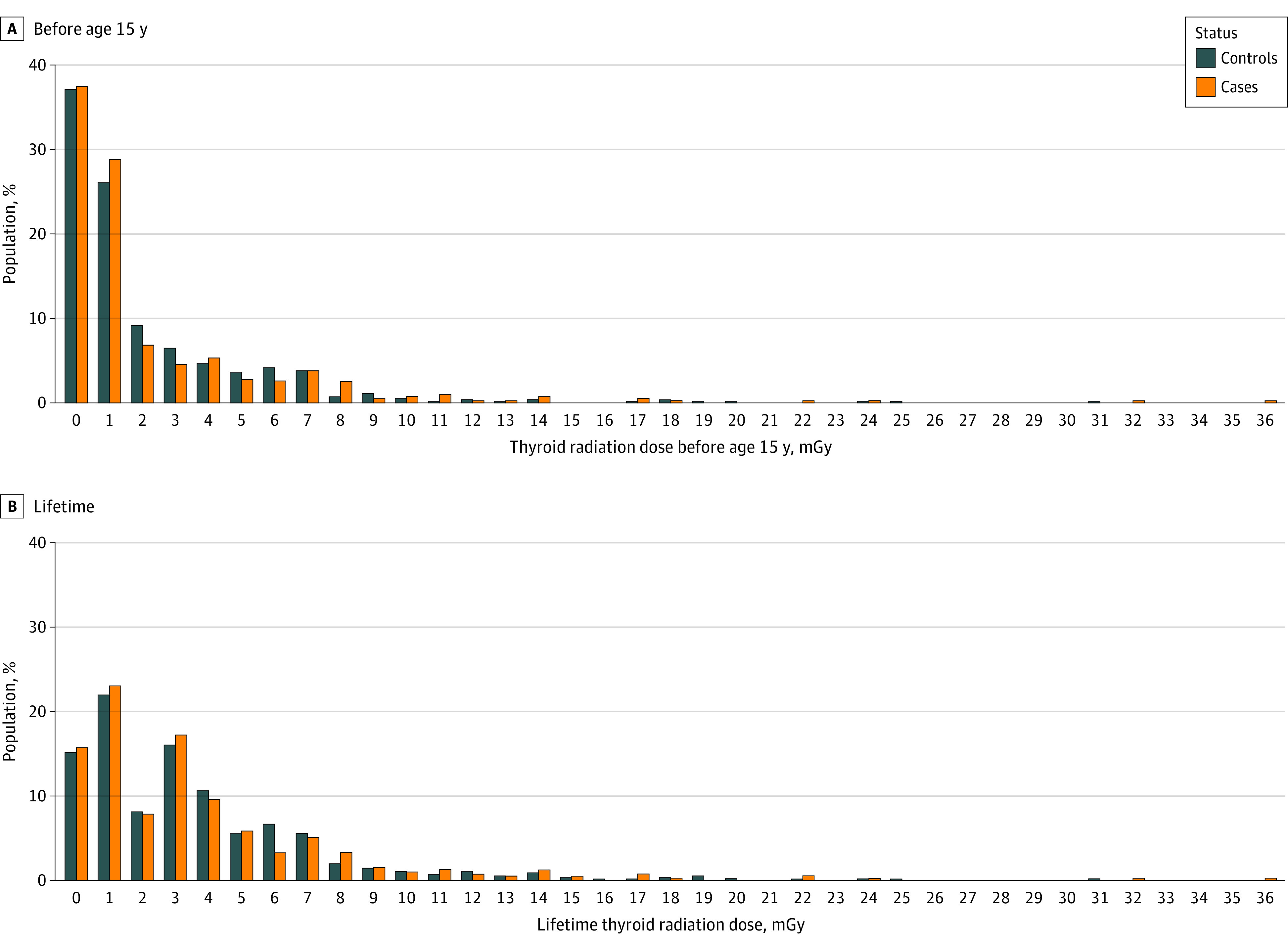
Radiation Dose to Thyroid From Atmospheric Nuclear Tests

The mean (range) lifetime thyroid dose received from exposure to radioactive fallout from the nuclear tests by study participants born in 1974 or before and living in FP at the time of the tests in 1966 to 1974, was 4.7 (0 to 36.3) mGy for cases and 4.6 (0 to 31.2) for controls. Most of the dose was received before age 15 years, when the mean (SD) thyroid doses received by cases and controls were 2.9 (0 to 36.3) mGy and 2.7 (0 to 31.2) mGy (Figure 1). Most of the dose was due to leafy vegetable consumption and was received in 1974 (eTable 5 in Supplement 1). Overall, the magnitude, timing, and sources of thyroid radiation dose were similar in the initial study and the extension (eTable 6 in Supplement 1).

### Epidemiological Analysis: Excess Relative Risk per Gray

**Figure 2.**
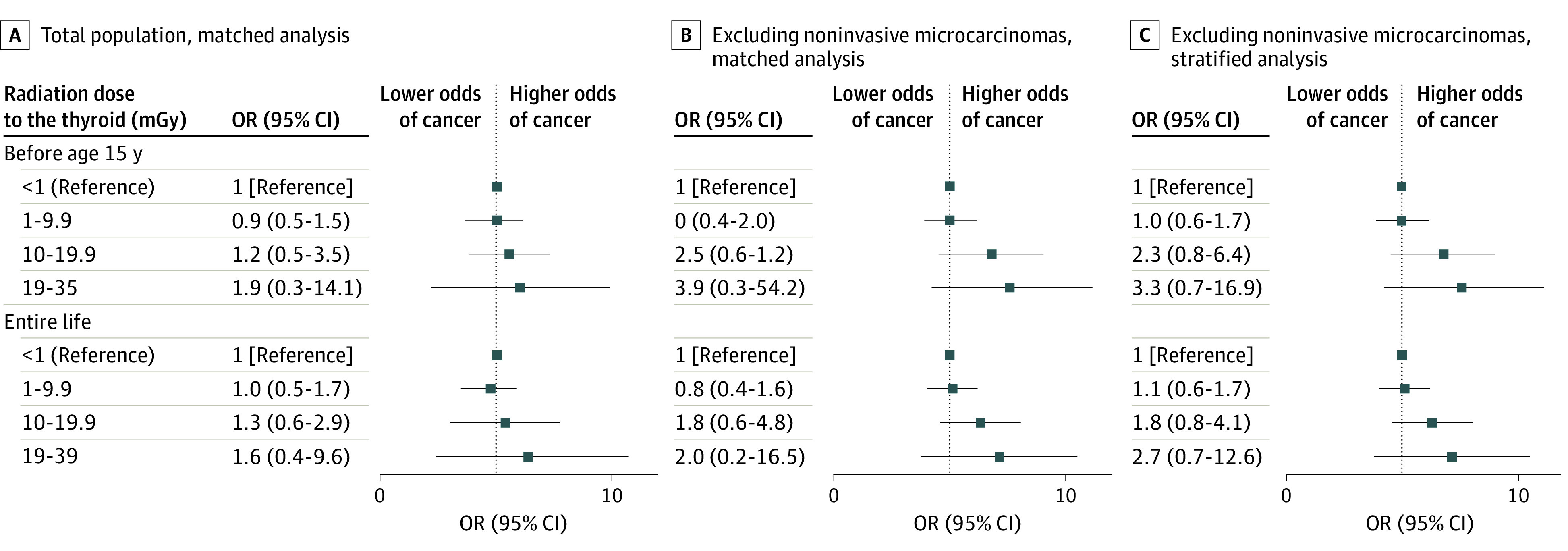
Thyroid Cancer Risk by Thyroid Dose Before Age 15 y and Lifetime Conditional (left and center) or unconditional (right) logistic regressions, adjusted by ethnic group, level of education, obesity, family history of thyroid cancer, personal history of radiotherapy, occupation at nuclear test site, dietary iodine intake, and number of pregnancies for women, are presented. OR indicates odds ratio.

Considering all thyroid cancers and any dose-response model, the risk of thyroid cancer was not found to increase significantly with the thyroid dose received before age 15 years (Figure 2; eTable 7 in Supplement 1); the excess relative risk (ERR) per milligray estimated by a linear model was 0.04 (95% CI, −0.09 to 0.17; *P* = .27). Excluding cases with unifocal microcarcinomas without extra thyroid extension and their controls, the radiation dose received to the thyroid before age 15 years was not associated with increased risk of thyroid carcinoma (ERR per milligray = 0.11; 95% CI, −0.15 to 0.36, *P* = .10). In unconditional multivariate regression excluding microcarcinomas but not their matched controls and stratified on sex and 5-year age class, the increase in risk was significant (ERR per milligray = 0.09; 95% CI, −0.03 to 0.20; *P* = .02). These results were similar when adjusting using a quadratic or an exponential dose-response model rather than a linear model (eTable 6 in Supplement 1). There were 13 cases (12 males and 1 female) and 14 controls (all females) who had worked at nuclear test sites during atmospheric tests, but this factor was not associated with increased risk of thyroid cancer (eTable 7 in Supplement 1). Similar results were observed when considering the lifetime thyroid dose due to nuclear tests rather than that received before age 15 years (Figure 2; eTable 8 in Supplement 1) and when restricting analyses to papillary thyroid carcinomas (eTables 9-10 in Supplement 1).

The ERR per milligray was significantly higher in inhabitants with parents of Polynesian origin (0.07; 95% CI, −0.10 to 0.25) than those with parents of other ethnic origins (−0.04; 95% CI, −0.11 to 0.25; *P* for interaction = .05). This interaction association was also found when excluding noninvasive microcarcinomas and their strata (Polynesian origin: ERR per milligray = 0.18; 95% CI, −0.18 to 0.55; other origins: −0.04; 955 CI, −0.19 to 0.23; *P* for interaction = .02). However, there was no interaction association in an unconditional logistic regression excluding noninvasive microcarcinomas (eTable 11 in Supplement 1).

### Epidemiological Analysis: Lifetime Thyroid Cancer Risk for Entire FP Population

**Figure 3.**
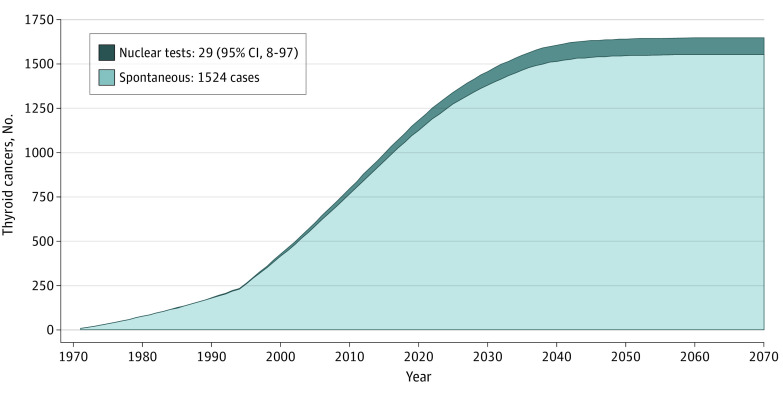
Estimated Number of Radiation-Induced Thyroid Cancers Spontaneous and nuclear radiation–induced thyroid cancers estimated in the cohort of inhabitants living in French Polynesia at the time of nuclear tests up to the extinction of the cohort are presented. The spontaneous number was derived from incidence rates published in *Cancer Incidence in Five Continents Volume VII*^21^ for calendar years before 1995 and *Cancer Incidence in Five Continents Volume IX* for 1995 and afterward.^22^ The Biological Effects of Ionizing Radiation (BEIR) VII Committee radiation risk model was used.^20^ For reasons of simplification, lifetime thyroid cancer risk was estimated under the assumption that all atmospheric nuclear tests took place in 1971.

The lifetime risk in the entire population present in FP during atmospheric nuclear tests was estimated to be an excess of 29 DTC cases (95% CI, 8-97 cases), or 2.3% (95% CI, 0.6%-7.7%) of 1524 spontaneous DTC cases expected from 1971 to 2070 in this population (Figure 3).^20,21,22^ Among these excess DTC cases, 17 DTC cases (95% CI, 5-58 cases) were estimated to have occurred before 2022, or 2.1% (95% CI, 0.6%-6.9%) of 1141 natural DTC cases estimated for this period. Of excess DTC cases, 27 cases (95% CI, 7-88 cases) were estimated to have occurred in females and 2 cases (95% CI, 1-7 cases) in males. In similar calculations using RadRAT software,^24^ Japanese life tables, and thyroid cancer incidence rates in 2010, we found a lifetime risk of 15 DTC cases (95% CI, 3-34 cases).

## Discussion

This case-control study of 395 cases and 555 controls investigated the association of the radiation dose received to the thyroid gland from radioactive fallout of nuclear atmospheric tests performed by France between 1966 and 1974 with the risk of DTC diagnosed between 1984 and 2016 in a population born in FP. We did not find a clear association between lifetime thyroid dose or the dose received before age 15 years and this risk. In unconditional multivariate regression excluding 137 noninvasive microcarcinomas but not their matched controls and stratified on sex and 5-year age class, the increase in risk was significant; however, it should be noted that the direction of the odds ratios were similar in entire sample and 2 sensitivity analyses. Based on a BEIR VII model,^20^ the lifetime risk in the entire population living in FP during atmospheric nuclear tests was estimated to be an excess of 29 DTC cases, or 2.3% of 1524 sporadic DTC cases expected in this population from 1971 to its extinction.

This study’s thyroid radiation dose estimates, which were based on original reports from internal radiation-protection services declassified by the French Army in 2013, were almost 2-fold higher than those estimated in the initial part of the case-control study, which were based on syntheses sent by the French Army to UNSCEAR. However, the entire case-control study risk estimates were slightly lower than those estimated in phase 1 of the study. This may be explained by a lower relative difference in thyroid dose estimates in DTC cases and controls in the entire study, likely associated with an improvement in the dose-reconstruction methodology arising from the use of focus groups and key informants. These methods were likely associated with better control of memory-recall biases.

Several points suggest that our results should be considered with caution. First, in the entire study, we did not confirm the association or dose-response modifiers (in particular, the number of pregnancies and obesity) found in the initial study. Second, using revised dose estimates, no significant dose response was found for the initial case-control study. This was true when considering all participants in the initial study, restricting the analysis to individuals with nonmicrocarcinomas and their matched controls, or restricting the analysis to individuals with nonmicrocarcinomas and all controls.

We estimated an ERR per milligray of 0.04 (95% CI,−0.09 to 0.17), or an ERR per gray of 40 (95% CI, −9 to 167), for risk of thyroid carcinoma when considering the thyroid radiation dose received before age 15 years. This was higher than but compatible with values of ERR per gray for exposure before age 15 years of 7.7 (95% CI, 2.1-28.7) estimated for external irradiation of thyroid in a meta-analysis of 7 studies^25^ and 4.5 (95% CI, 2.1-8.5) and 7.4 (95% CI, 3.1-16.3) estimated using different models after the Chernobyl accident.^26^ Additionally, low and moderate thyroid radiation doses (<100 mGy) may be proportionally more carcinogenic than doses higher than several grays because of a lack of cell killing.^27,28,29^

Our results showed that having worked on the nuclear sites was not associated with increased risk of thyroid cancer, a finding based on only 13 cases. This was not surprising given that most thyroid cancers occurred in women, whereas almost all atmospheric nuclear tests participants were men.

### Limitations

Our study has several limitations. Most importantly, although we included most cases of thyroid cancer diagnosed since 1983 in FP, the power of our study was very low owing to the very low level of thyroid radiation dose. Another major limitation of this study is that, opposite to what we did for phase 1 of the study, we were not able to obtain access to data of the Cancer Registry of FP for phase 2, which therefore included only thyroid cancers treated in public or private hospitals or by private endocrinologists. Therefore, our study could have missed some cases, for example, individuals diagnosed and treated outside FP. However, in preparing the initial case-control study, we conducted extensive investigation and contacted hospitals in France, Australia, and New Zealand known to treat FP individuals. We found that virtually all patients with thyroid cancer treated outside FP were also registered for their cancer at the only public hospital in FP that participated in both the initial study and the present study. Therefore, the number of thyroid cancer cases that were not included in the study was likely small. It must be noted that the Cancer Registry of FP did not provide data after those published by the International Agency for Research on Cancer in 2007^22^ given that data published in the Global Cancer Observatory 2020^30^ were the same as those published in 2007. Additionally, with the exception of those published by the principal investigator of this study, to our knowledge, there have been no peer-reviewed publications on the data of this registry, which was created by the South Pacific Commission in 1983.^31^

## Conclusion

In this case-control study of 395 cases and 555 controls, we found that there was no clear association between French nuclear tests and increased risk of DTC in FP residents. However, the low level of thyroid radiation dose estimates and several inconsistencies in results may limit the interpretability of our results. According to current knowledge on radiation-induced thyroid cancer, between 0.6% and 7.7% of thyroid cancers occurring in inhabitants living in FP at the time of nuclear tests may have been associated with nuclear tests. These findings suggest that there is a need for further epidemiological surveillance, which may require a quality cancer registry open to scientific researchers.

## Notes

Supplement 1. **eTable 1.** Description of Thyroid Cancer Cases by Phase of Study **eTable 2.** Population Main Residence in French Polynesia at Time of Census of 1971 **eTable 3.** Mean Thyroid Radiation Dose Estimated for Controls by Age Group and Archipelago of Main Residence, 1971 **eTable 4.** Characteristics of Cases and Controls, by Phase of Study **eTable 5.** Characteristics of Thyroid Radiation Dose From Atmospheric Nuclear Tests Among Individuals Born 1974 or Before Present in French Polynesia at Time of Tests **eTable 6.** Characteristics of Thyroid Radiation Dose From Atmospheric Nuclear Tests Among Individuals Born 1974 or Before and Present in French Polynesia at Time of Tests by Phase of Study **eTable 7.** Thyroid Cancer Risk by Radiation Dose Received to Thyroid Before Age 15 y and by Nuclear Site Work Status **eTable 8.** Thyroid Cancer Risk by Lifetime Thyroid Radiation Dose and Nuclear Site Work Status **eTable 9.** Papillary Differentiated Thyroid Carcinoma Risk by Radiation Dose Received to Thyroid Before Age 15 y and Nuclear Site Work Status **eTable 10.** Papillary Differentiated Thyroid Carcinoma Risk by Lifetime Thyroid Radiation Dose and Nuclear Site Work Status **eTable 11.** Radiation Dose Response Modifiers for Dose Received Before Age 15 y **eFigure.** Study Flowchart

Supplement 2.
Data Sharing Statement
