# Supplementary material for: Assessment of Differentiated Thyroid Carcinomas in French Polynesia After Atmospheric Nuclear Tests Performed by France
Source: JAMA Netw Open. 2023 May 5;6(5):e2311908. doi: 10.1001/jamanetworkopen.2023.11908 (PMC10163383; doi:10.1001/jamanetworkopen.2023.11908)
Supplement: Supplement 1. — eTable 1. Description of Thyroid Cancer Cases by Phase of Study eTable 2. Population Main Residence in French Polynesia at Time of Census of 1971 eTable 3. Mean Thyroid Radiation Dose Estimated for Controls by Age Group and Archipelago of Main Residence, 1971 eTable 4. Characteristics of Cases and Controls, by Phase of Study eTable 5. Characteristics of Thyroid Radiation Dose From Atmospheric Nuclear Tests Among Individuals Born 1974 or Before Present in French Polynesia at Time of Tests eTable 6. Characteristics of Thyroid Radiation Dose From Atmospheric Nuclear Tests Among Individuals Born 1974 or Before and Present in French Polynesia at Time of Tests by Phase of Study eTable 7. Thyroid Cancer Risk by Radiation Dose Received to Thyroid Before Age 15 y and by Nuclear Site Work Status eTable 8. Thyroid Cancer Risk by Lifetime Thyroid Radiation Dose and Nuclear Site Work Status eTable 9. Papillary Differentiated Thyroid Carcinoma Risk by Radiation Dose Received to Thyroid Before Age 15 y and Nuclear Site Work Status eTable 10. Papillary Differentiated Thyroid Carcinoma Risk by Lifetime Thyroid Radiation Dose and Nuclear Site Work Status eTable 11. Radiation Dose Response Modifiers for Dose Received Before Age 15 y eFigure. Study Flowchart [file jamanetwopen-e2311908-s001.pdf]

Supplemental Online Content

de Vathaire F, Zidane M, Xhaard C, et al. Assessment of differentiated thyroid carcinomas in French Polynesia after atmospheric nuclear tests performed by France. *JAMA Netw Open*. 2023;6(5):e2311908. doi:10.1001/jamanetworkopen.2023.11908

eTable 1. Description of Thyroid Cancer Cases by Phase of Study

eTable 2. Population Main Residence in French Polynesia at Time of Census of 1971

eTable 3. Mean Thyroid Radiation Dose Estimated for Controls by Age Group and Archipelago of Main Residence, 1971

eTable 4. Characteristics of Cases and Controls, by Phase of Study

eTable 5. Characteristics of Thyroid Radiation Dose From Atmospheric Nuclear Tests Among Individuals Born 1974 or Before Present in French Polynesia at Time of Tests

eTable 6. Characteristics of Thyroid Radiation Dose From Atmospheric Nuclear Tests Among Individuals Born 1974 or Before and Present in French Polynesia at Time of Tests by Phase of Study

eTable 7. Thyroid Cancer Risk by Radiation Dose Received to Thyroid Before Age 15 y and by Nuclear Site Work Status

eTable 8. Thyroid Cancer Risk by Lifetime Thyroid Radiation Dose and Nuclear Site Work Status

eTable 9. Papillary Differentiated Thyroid Carcinoma Risk by Radiation Dose Received to Thyroid Before Age 15 y and Nuclear Site Work Status

eTable 10. Papillary Differentiated Thyroid Carcinoma Risk by Lifetime Thyroid Radiation Dose and Nuclear Site Work Status

eTable 11. Radiation Dose Response Modifiers for Dose Received Before Age 15 y

eFigure. Study Flowchart

This supplemental material has been provided by the authors to give readers additional information about their work.

© 2023 de Vathaire F et al. *JAMA Network Open.*

eTable 1. Description of Thyroid Cancer Cases by Phase of Study

	Phase 1 (n=229)	Phase 2 (n=166)
Women (%)	88.6	81.1
Age at time of first test (years)		
Born after tests	4.8	25.3
Born during tests	11.8	21.0
0-14	52.0	40.4
15+	31.4	13.3
Age at diagnosis (years)		
10-19	3.5	3.0
20-29	17.0	4.8
30-39	33.2	14.4
40-49	27.5	25.3
50-62	18.8	52.4
Histology : % papillary	76.9	83.7
Tumour size (mm)		
≤ 10	52.2	48.0
11-40	36.9	41.3
>40	10.8	10.7
Multifocal tumour (%)	39.0	35.7
Bilateral tumour (%)	22.2	25.0
Unifocal microcarcinoma (%)	35.8	33.1

© 2023 de Vathaire F et al. *JAMA Network Open.*

eTable 2. Population Main Residence in French Polynesia at Time of Census of 1971

Gender	Age group	Windward Islands	Leeward Islands	Marquisas Islands	Autrales Islands	Tuamotu-Gambier archipelago	Whole French Polynesia
Men	0	7008	1466	594	474	657	10199
Men	5	6111	1312	582	422	562	8989
Men	10	5112	1201	482	381	411	7587
Men	15	4834	927	337	315	362	6775
Men	20	5029	469	124	137	572	6331
Men	25	3415	409	142	130	380	4476
Men	30	2960	433	143	122	364	4022
Men	35	2824	395	138	137	298	3792
Men	40	2266	361	111	101	264	3103
Men	45	1763	277	72	102	212	2426
Men	50	1182	230	72	80	176	1740
Men	55	1052	274	47	64	145	1582
Men	60	762	217	61	52	86	1178
Men	65	555	146	33	40	67	841
Men	70	294	79	14	31	36	454
Men	75	153	38	13	12	17	233
Men	80	69	17	5	8	14	113
Men	85	36	15	3	2	2	58
Women	0	6390	1412	555	491	595	9443
Women	5	5956	1308	497	485	522	8768
Women	10	4820	1233	454	374	411	7292
Women	15	4411	969	366	302	334	6382
Women	20	3072	432	159	133	209	4005
Women	25	2940	406	139	130	200	3815
Women	30	2685	398	133	131	180	3527
Women	35	2452	350	130	115	154	3201
Women	40	1903	318	90	107	132	2550
Women	45	1543	310	71	82	139	2145
Women	50	1026	227	49	62	115	1479
Women	55	987	216	55	50	110	1418
Women	60	722	189	36	39	74	1060
Women	65	474	128	20	29	54	705
Women	70	240	41	15	21	27	344
Women	75	137	31	8	10	16	202
Women	80	71	12	2	5	5	95
Women	85	41	7	2	2	1	53

© 2023 de Vathaire F et al. *JAMA Network Open.*

eTable 3. Mean Thyroid Radiation Dose Estimated for Controls by Age Group and Archipelago of Main Residence, 1971

Age group in 1971	Windward Islands	Leeward Islands	Marquisas Islands	Autrales Islands	Tuamotu-Gambier archipelago
0	4.58	1.41	0.02	10.63	0.24
5	6.61	2.88	0.89	9.09	5.43
10	5.85	2.44	1.3	8.24	4.17
15	5.22	2.47	1.8	5.32	3.72
20	4.54	0.68	2.02	3.41	3.58
25	4.65	2.05	0.38	3.99	3.98

© 2023 de Vathaire F et al. *JAMA Network Open.*

eTable 4. Characteristics of Cases and Controls, by Phase of Study

	Phase 1			Phase 2			P-value[a]
Characteristic	Cases (n=229)	Controls (n=373)	P-value	Cases (n=166)	Controls (n=182)	P-value	(initial vs extension)
Women (%)	88.6	87.4	-[b]	80.2	80.8	-[b]	0.002
Age at end of follow-up (years) : mean (std)	38.5 (10.4)	38.3 (10.2)	-[b]	50.0 (13.2)	50.2 (13.1)	-[b]	<0.0001
Parents Ethnical origin (%)							
Both Polynesian	53.5	51.0		58.4	42.3		
Polynesian- Asian	14.0	11.3	0.2	15.0	18.7	0.05	0.06
Polynesian-European	20.2	19.3		15.0	17.0		
Polynesian-Asian-European / Other	12.3	14.2		11.2	22.0		
Familial history of thyroid cancer (%)	5.2	1.9	0.03	4.8	1.1	0.08	0.4
Familial history of thyroid pathology (%)	40.6	15.8	<0.0001	37.4	20.3	0.0004	0.09
Personal history of radiation therapy (%)	3.5	0.5	<0.01	15.7	1.7	0.0006	<0.0001
Location of birth (%)							
Tahiti & Moorea	55.5	57.4		63.3	57.8		

Other Société islands	20.5	18.2	0.9	19.9	18.9	0.5	0.1
Australes	6.1	4.8		6.6	6.7		
Marquises	6.1	5.9		2.4	2.8		
Tuamotu-Gambier	11.8	13.7		13.5	8.3		
Mean height (m)							
Women	1.64	1.63	0.002	1.66	1.65	0.3	<0.001
Men	1.75	1.73		176	1.75		
Mean weight at end follow-up (kg)							
Women	82	73	<0.0001	86	78	0.0004	<0.0001
Men	98	89		106	93		
Mean BMI at end follow-up							
Women	30.1	27.5	<0.0001	31.5	28.9	0.001	0.0004
Men	31.9	29.8		34.4	30.2		
Diploma (%)							
No	44.1	37.0	0.06	36.6	32.4	0.4	<0.0001
Yes	55.9	63.0		63.9	67.6		

© 2023 de Vathaire F et al. *JAMA Network Open.*

Number of pregnancies (%)							
0	12.3	15.0		12.2	12.2		
1-4	44.8	51.5	0.01	53.4	65.3	0.02	<0.0001
5-16	42.9	33.4		35.4	22.5		

[a] Overall comparison between initial and extension study, stratified on the status (case/control)

[b] Matching criteria

© 2023 de Vathaire F et al. *JAMA Network Open.*

eTable 5. Characteristics of Thyroid Radiation Dose From Atmospheric Nuclear Tests Among Individuals Born 1974 or Before Present in French Polynesia at Time of Tests

Dose in mGy mean, median (min-max)	Thyroid cancer cases (n=337)	Controls (n=441)
Lifetime dose		
Whole sample	4.71; 3.47 (<0.01-36.25)	4.60; 3.65 (<0.01-31.19)
Gender		
Male	4.32; 3.28 (<0.01-17.89)	4.29; 3.27 (<0.01-22.15)
Female	4.77; 3.54 (<0.01-36.25)	4.65; 3.66 (<0.01-31.19)
Source		
Leafy vegetables	3.28; 2.59 (<0.01-23.01)	3.30; 2.55 (<0.01-29.63)
Fresh cow's milk*	0.58; 0.02 (<0.01-17.65)	0.56; 0.09 (<0.01-8.92)
Inhalation	0.22; 0.16 (<0.01-4.64)	0.19; 0.16 (<0.01-1.63)
Cistern drinking water	0.16; <0.01 (<0.01-6.52)	0.11; <0.01 (<0.01-5.64)
Other foodstuffs	0.04; 0.02 (<0.01-0.35)	0.05; 0.02 (<0.01-0.94)
External irradiation	0.43; 0.43 (<0.01-5.81)	0.39; 0.44 (<0.01-3.5)
Year of exposure		
1966	0.8; 0.33 (0.02-29.82)	0.48; 0.33 (0.01-9.03)
1967	0.99; 0.7 (0.04-5.31)	0.99; 0.71 (0.02-7.2)
1968	0.29; 0.22 (<0.01-1.73)	0.29; 0.22 (0.01-1.38)
1969	0.01; 0.01 (<0.01-0.06)	0.01; 0.01 (<0.01-0.08)
1970	0.15; 0.11 (0.01-0.97)	0.15; 0.12 (0.01-0.82)
1971	0.38; 0.26 (<0.01-4.17)	0.36; 0.26 (<0.01-2.67)
1972	0.06; 0.05 (<0.01-0.41)	0.06; 0.04 (<0.01-0.36)

© 2023 de Vathaire F et al. *JAMA Network Open.*

1973	0.32; 0.07 (<0.01-22.6)	0.33; 0.07 (<0.01-14.83)
1974	2.28; 1.89 (<0.01-20.19)	2.44; 1.9 (<0.01-21.52)
Location of residence at time of birth		
Tahiti & Moorea	4.90; 4.06 (<0.01-24.29)	5.12; 4.14 (0-25.12)
Other Société islands	3.63; 3.03 (0.21-17.89)	3.54; 2.74 (0.18-31.19)
Marquises	3.43; 1.03 (0.02-32.1)	1.99; 1.14 (0.03-7)
Australes	6.85; 5.41 (0.67-22.79)	7.37; 5.15 (1.78-18.29)
Tuamotu-Gambier	5.65; 3.46 (0.02-36.25)	3.78; 3.5 (<0.01-14.64)
Dose received before the age of 15		
Whole sample	2.93; 1.30 (<0.01-36.15)	2.71; 1.50 (<0.01-31.19)
Gender		
Male	2.61; 1.16 (<0.01-17.89)	2.15; 1.03 (<0.01-11.87)
Female	2.99; 1.32 (<0.01-36.15)	2.81; 1.56 (<0.01-31.19)
Source		
Leafy vegetables	1.95; 0.92 (<0.01-23.01)	1.84; 0.87 (<0.01-29.63)
Fresh cow's milk*	0.43; <0.01 (<0.01-17.65)	0.42; 0 (<0.01-8.92)
Inhalation	0.16; 0.06 (<0.01-4.64)	0.13; 0.07 (<0.01-0.75)
Cistern drinking water	0.13; <0.01 (<0.01-6.52)	0.09; <0.01 (<0.01-5.64)
Other foodstuffs	0.02; <0.01 (<0.01-0.24)	0.02; <0.01 (<0.01-0.29)
External irradiation	0.25; 0.09 (<0.01-5.8)	0.22; 0.10 (<0.01-2.25)
Year of exposure		
1966	0.63; 0.24 (<0.01-29.82)	0.35; 0.25 (<0.01-3.21)
1967	0.71; 0.47 (<0.01-5.31)	0.72; 0.44 (<0.01-7.2)
1968	0.21; 0.13 (0-1.73)	0.20; 0.12 (<0.01-1.38)

© 2023 de Vathaire F et al. *JAMA Network Open.*

1969	0.01; <0.01 (<0.01-0.06)	<0.01; <0.01 (<0.01-0.04)
1970	0.1; 0.06 (<0.01-0.97)	0.09; 0.05 (<0.01-0.82)
1971	0.24; 0.05 (0-40.17)	0.22; 0.06 (<0.01-2.67)
1972	0.03; <0.01 (<0.01-0.41)	0.03; <0.01 (<0.01-0.36)
1973	0.27; <0.01 (<0.01-22.6)	0.22; <0.01 (<0.01-14.83)
1974	1.16; <0.01 (<0.01-20.19)	1.24; <0.01 (<0.01-21.52)
Location of residence at time of birth		
Tahiti & Moorea	3.18; 1.86 (<0.01-24.29)	3.15; 1.98 (<0.01-25.12)
Other Société islands	1.87; 1.17 (<0.01-17.89)	1.99; 0.86 (<0.01-31.19)
Marquises	2.10; 0.34 (<0.01-32.1)	0.72; 0.33 (<0.01-3.77)
Australes	3.63; 0.68 (<0.01-22.79)	3.44; 1.03 (<0.01-18.29)
Tuamotu-Gambier	3.99; 1.18 (<0.01-36.15)	2.35 0.91 (<0.01-14.64)

© 2023 de Vathaire F et al. *JAMA Network Open.*

eTable 6. Characteristics of Thyroid Radiation Dose From Atmospheric Nuclear Tests Among Individuals Born 1974 or Before and Present in French Polynesia at Time of Tests by Phase of Study

		Phase 1		Phase 2	
Dose in mGy mean, median (min-max)		Thyroid cancer cases (n=229)	Controls (n=373)	Thyroid cancer cases (n=166)	Controls (n=182)
Lifetime dose					
Whole sample		4.77; 3.84 (<0.01-32.1)	5.03; 4.02 (<0.01-25.12)	4.59; 3.04 (0.02-36.25)	3.36; 2.17 (<0.01-31.19)
Gender					
	Male	4.27; 3.33 (<0.01-15.71)	5.26; 4.82 (0.01-22.15)	4.37; 3.15 (0.23-17.89)	2.56; 1.56 (<0.01-8.25)
	Female	4.83; 3.88 (<0.01-32.1)	5.00; 3.99 (<0.01-25.12)	4.65; 3.02 (0.02-36.25)	3.56; 2.31 (0.03-31.19)
Source					
	Leafy vegetables	3.49; 2.61 (<0.01-23.01)	3.76; 2.67 (<0.01-21.54)	2.88; 1.91 (<0.01-20.35)	1.98; 0.87 (<0.01-29.63)
	Fresh cow's milk [a]	0.48; 0.01 (<0.01-17.65)	0.51; 0.04 (<0.01-8.92)	0.77; 0.02 (<0.01-12.19)	0.7; 0.15 (<0.01-5.92)
	Inhalation	0.2; 0.15 (<0.01-4.64)	0.18; 0.16 (<0.01-1.63)	0.26; 0.16 (0.01-3.57)	0.21; 0.17 (<0.01-0.75)
	Cistern drinking water	0.13; <0.01 (<0.01-6.02)	0.11; 0 (<0.01-4.56)	0.21; <0.01 (<0.01-6.52)	0.1; <0.01 (<0.01-5.64)
	Other foodstuffs	0.05; 0.03 (<0.01-0.35)	0.06; 0.03 (<0.01-0.94)	0.03; 0.01 (<0.01-0.34)	0.02; 0.01 (<0.01-0.14)

© 2023 de Vathaire F et al. *JAMA Network Open.*

External irradiation	0.42; 0.44 (<0.01-4.59)	0.4; 0.44 (<0.01-3.5)	0.44; 0.3 (0.01-5.81)	0.35; 0.32 (<0.01-1.47)
Year of exposure				
1966	0.58; 0.32 (0.05-13.59)	0.53; 0.36 (0.05-9.03)	1.29; 0.34 (0.02-29.82)	0.30; 0.20 (0.01-1.20)
1967	0.96; 0.69 (0.10-5.31)	1.06; 0.76 (0.05-6.79)	1.07; 0.82 (0.04-4.45)	0.74; 0.39 (0.02-7.20)
1968	0.30; 0.23 (0.03-1.73)	0.32; 0.23 (0.03-1.38)	0.27; 0.19 (<0.01-1.28)	0.17; 0.11 (0.01-1.30)
1969	0.01; 0.01 (<0.01-0.06)	0.01; 0.01 (<0.01-0.08)	0.01; 0.01 (<0.01-0.04)	0.01; 0.01 (<0.01-0.02)
1970	0.15; 0.11 (0.01-0.97)	0.16; 0.13 (0.02-0.68)	0.14; 0.11 (0.01-0.68)	0.11; 0.07 (0.01-0.82)
1971	0.38; 0.27 (0.01-3.61)	0.4; 0.28 (0.01-2.04)	0.38; 0.24 (<0.01-4.17)	0.26; 0.16 (<0.01-2.67)
1972	0.06; 0.05 (<0.01-0.38)	0.06; 0.05 (<0.01-0.3)	0.05; 0.04 (<0.01-0.41)	0.04; 0.03 (<0.01-0.36)
1973	0.41; 0.07 (<0.01-22.6)	0.36; 0.07 (<0.01-14.83)	0.16; 0.06 (<0.01-6.29)	0.26; 0.05 (<0.01-13.77)
1974	2.38; 2.00 (<0.01-20.19)	2.61; 2.07 (<0.01-18.05)	2.10; 1.20 (<0.01-14.78)	1.95; 1.22 (<0.01-21.52)
Archipelago of residence at time of birth				
Tahiti & Moorea	4.97; 4.25 (<0.01-24.29)	5.68; 4.47 (<0.01-25.12)	4.76; 3.65 (0.02-14.95)	3.62; 2.46 (<0.01-9.71)
Other Société islands	3.79; 3.17 (0.67-15.71)	3.69; 3.22 (0.99-20.31)	3.38; 2.33 (0.21-17.89)	3.2; 1.22 (0.18-31.19)
Marquises	4.20; 1.23 (0.02-32.1)	2.47; 2.15 (0.38-7)	2.22; 1.03 (0.19-7.45)	0.55; 0.41 (0.03-1.23)
Australes	7.66; 5.56 (1.26-22.79)	7.54; 5.15 (2.67-18.29)	4.01; 3.39 (0.67-8.6)	6.42; 5.07 (1.78-13.79)

© 2023 de Vathaire F et al. *JAMA Network Open.*

Tuamotu-Gambier	4.36; 3.44 (0.02-18.26)	4.07; 3.67 (<0.01-14.64)	7.95; 3.65 (0.23-36.25)	2.35; 1.2 (0.36-7.86)
Dose received before 15 years				
Whole sample	2.69; 1.2 (<0.01-32.1)	2.7; 1.51 (<0.01-25.12)	3.38; 1.42 (<0.01-36.15)	2.74; 1.45 (<0.01-31.19)
Gender				
Male	1.87; 1.15 (<0.01-8.22)	2.29; 1.15 (<0.01-11.87)	3.35; 1.42 (<0.01-17.89)	1.89; 0.91 (<0.01-8.25)
Female	2.8; 1.28 (<0.01-32.1)	2.76; 1.59 (<0.01-25.12)	3.38; 1.43 (<0.01-36.15)	2.96; 1.5 (<0.01-31.19)
Source				
Leafy vegetables	1.91; 0.92 (<0.01-23.01)	1.91; 0.99 (<0.01-21.54)	2.01; 1.02 (<0.01-20.28)	1.63; 0.52 (<0.01-29.63)
Fresh cow's milk [a]	0.30; <0.01 (<0.01-17.65)	0.36; 0 (<0.01-8.92)	0.67; <0.01 (<0.01-12.19)	0.57; 0.01 (<0.01-5.92)
Inhalation	0.13; 0.05 (<0.01-4.64)	0.11; 0.06 (<0.01-0.72)	0.20; 0.09 (<0.01-3.56)	0.17; 0.10 (<0.01-0.75)
Cistern drinking water	0.11; <0.01 (<0.01-6.02)	0.09; <0.01 (<0.01-4.56)	0.17; <0.01 (<0.01-6.52)	0.10; <0.01 (<0.01-5.64)
Other foodstuffs	0.01; <0.01 (<0.01-0.24)	0.02; <0.01 (<0.01-0.29)	0.02; <0.01 (<0.01-0.14)	0.01; <0.01 (<0.01-0.12)
External irradiation	0.22; 0.09 (<0.01-4.04)	0.21; 0.09 (<0.01-2.25)	0.31; 0.12 (<0.01-5.8)	0.26; 0.14 (<0.01-1.47)
Year of exposure				
1966	0.46; 0.25 (<0.01-13.59)	0.37; 0.28 (<0.01-3.21)	1; 0.23 (<0.01-29.82)	0.26; 0.18 (<0.01-1.2)
1967	0.64; 0.40 (<0.01-5.31)	0.73; 0.49 (<0.01-6.79)	0.85; 0.52 (<0.01-3.85)	0.66; 0.29 (<0.01-7.2)

	1968	0.2; 0.12 (<0.01-1.73)	0.21; 0.15 (<0.01-1.38)	0.22; 0.13 (<0.01-1.08)	0.15; 0.08 (<0.01-1.3)
	1969	<0.01; <0.01 (<0.01-0.06)	<0.01; <0.01 (<0.01-0.04)	0.01; <0.01 (<0.01-0.02)	<0.01; <0.01 (<0.01-0.01)
	1970	0.09; 0.03 (<0.01-0.97)	0.1; 0.05 (<0.01-0.68)	0.11; 0.07 (<0.01-0.68)	0.09; 0.05 (<0.01-0.82)
	1971	0.22; <0.01 (<0.01-3.61)	0.22; 0.04 (<0.01-2.04)	0.28; 0.10 (<0.01-4.17)	0.21; 0.09 (<0.01-2.67)
	1972	0.03; <0.01 (<0.01-0.38)	0.03; <0.01 (<0.01-0.3)	0.04; 0.02 (<0.01-0.41)	0.03; 0.02 (<0.01-0.36)
	1973	0.35; <0.01 (<0.01-22.6)	0.21; <0.01 (<0.01-14.83)	0.13; 0.02 (<0.01-6.29)	0.24; 0.02 (<0.01-13.77)
	1974	1.01; <0.01 (<0.01-20.19)	1.15; <0.01 (<0.01-18.05)	1.44; 0.05 (<0.01-14.78)	1.52; 0.17 (<0.01-21.52)
Archipelago of residence at time of birth					
	Tahiti & Moorea	2.83; 1.74 (<0.01-24.29)	3.25; 2.12 (<0.01-25.12)	3.86; 2.85 (<0.01-14.95)	2.9; 1.87 (<0.01-9.71)
	Other Société islands	1.6; 1.17 (<0.01-8.39)	1.74; 0.99 (<0.01-20.31)	2.28; 1.18 (<0.01-17.89)	2.58; 0.71 (<0.01-31.19)
	Marquises	2.64; 0.35 (<0.01-32.1)	0.78; 0.31 (<0.01-3.77)	1.24; 0.26 (<0.01-6.69)	0.53; 0.34 (0.03-1.23)
	Australes	4.29; 0.92 (<0.01-22.79)	2.89; 0.33 (<0.01-18.29)	1.35; 0.03 (<0.01-5.34)	6.42; 5.07 (1.78-13.79)
	Tuamotu-Gambier	3.19; 1.3 (<0.01-18.26)	2.44; 1.33 (<0.01-14.64)	5.43; 0.66 (<0.01-36.15)	1.89; 0.77 (<0.01-7.85)

[a]Only in Tahiti.

© 2023 de Vathaire F et al. *JAMA Network Open.*

eTable 7. Thyroid Cancer Risk by Radiation Dose Received to Thyroid Before Age 15 y and by Nuclear Site Work Status

Exposure	Total population, matched analysis (395 cases and 555 controls)			Excluding unifocal micro carcinomas size and their controls, matched analysis (258 cases / 359 controls)			Excluding unifocal micro carcinomas, unmatched analysis, stratified on study, sex and age (258 cases / 555 controls)		
	Cases/ Controls	OR (95%CI) [a]	p-value	Cases/ Controls	OR (95%CI)[a]	p-value	Cases/ Controls	OR (95%CI)[a]	p-value
Thyroid dose before age 15									
<1 mGy	203/272	1[b]		133/180	1 [b]		203/272	1 [b]	
1– 9.9 mGy	173/266	0.9 (0.5; 1.5)		107/170	0.9 (0.4; 2.0)		107/266	1.0 (0.6; 1.7)	
10 – 19.9 mGy	15/13	1.2 (0.4; 3.5)		14/7	2.5 (0.6; 10.2)		14/13	2.3 (0.8; 6.4)	
20 – 35 mGy	4/4	1.9 (0.3; 14.1)		4/2	3.9 (0.3; 54.2)		4/4	3.3 (0.7; 16.9)	
Linear model		0.040 (-0.086; 0.17)	0.3		0.11 (-0.15; 0.36)	0.095		0.085 (-0.033; 0.20)	0.02
Exponential model		0.078 (-0.015; 0.017)	0.19		0.15 (0.047; 0.26)	0.02		0.083 (0.025; 0.14)	0.02
Quadratic model		0.0050 (-0.0060; 0.016)	0.13		0.015 (-0,012; 0.041)	0.02		0.0070 (-0.0020; 0.021)	0.01

© 2023 de Vathaire F et al. *JAMA Network Open.*

Work at nuclear sites during tests									
No	375/541	1[b]		248/351	1[b]		248/541	1[b]	
Yes	13/14	1.4 (0.5; 4.1)	0.5	10/8	1.5 (0.5; 5.1)	0.5	10/14	2.0 (0.6; 6.3)	0.2

[a]OR: odds ratio, CI: confidence interval. Adjusted for ethnic group, level of education, obesity, family history of thyroid cancer, personal history of radiotherapy, dietary iodine intake, and number of pregnancies for women.

[b]Reference category

.

© 2023 de Vathaire F et al. *JAMA Network Open.*

eTable 8. Thyroid Cancer Risk by Lifetime Thyroid Radiation Dose and Nuclear Site Work Status

Exposure	Total population, matched analysis (395 cases and 555 controls)			Excluding unifocal micro carcinomas size and their controls, matched analysis (258 cases / 359 controls)			Excluding unifocal micro carcinomas, unmatched analysis, stratified on study, sex and age (258 cases / 555 controls)		
	Cases/ Controls	OR (95%CI)[a]	p-value	Cases/ Controls	OR (95%CI)[a]	p-value	Cases/ Controls	OR (95%CI)[a]	p-value
Lifetime thyroid dose									
<1 mGy	106/132	1[b]		78/96	1 [b]		78/132	1 [b]	
1– 9.9 mGy	259/385	1.0 (0.54; 1.68)	0.2	156/243	0.83 (0.42; 1.64)	0.3	156/385	1.05 (0.64; 1.73)	0.2
10 – 19.9 mGy	25/33	1.3 (0.59; 2.85)		20/17	1.75 (0.64; 4.78)		20/33	1.83 (0.82; 4.08)	
20 – 39 mGy	5/4	1.6 (0.28; 9.57)		4/3	1.96 (0.23; 16.5)		4/4	2.69 (0.57; 12.6)	
Linear model		0.051 (-0.058; 0.16)	0.2		0.10 (-0.098; 0.30)	0.07		0.058 (-0.029; 0.14)	0.05
Exponential model		0.065 (-0.013; 0.14)	0.2		0.12 (0.038; 0.21)	0.02		0.068 (0.0013; 0.012)	0.04

© 2023 de Vathaire F et al. *JAMA Network Open.*

Quadratic model		0.0033 (-0.0035; 0.010)	0.2		0.011 (-0,0063; 0.027)	0.02		0.0038 (-0.0015; 0.0092)	0.04
Work at nuclear sites during tests									
No	375/541	1[b]		248/351	1[b]		248/541	1[b]	
Yes	13/14	1.45 (0.49; 4.25)	0.5	10/8	1.63 (0.47; 5.64)	0.4	10/14	1.92 (0.61; 6.01)	0.3

[a]OR: odds ratio, CI: confidence interval. Adjusted for ethnic group, level of education, obesity, family history of thyroid cancer, personal history of radiotherapy, dietary iodine intake, and number of pregnancies for women.

[b]Reference category

© 2023 de Vathaire F et al. *JAMA Network Open.*

eTable 9. Papillary Differentiated Thyroid Carcinoma Risk by Radiation Dose Received to Thyroid Before Age 15 y and Nuclear Site Work Status

Exposure	Total population, matched analysis (315 cases and 438 controls)			Excluding unifocal micro carcinomas size and their controls, matched analysis (198 cases / 273 controls)			Excluding unifocal micro carcinomas, unmatched analysis, stratified on study, sex and age (198 cases / 555 controls)		
	Cases/ Controls	OR (95%CI) [a]	p-value	Cases/ Controls	OR (95%CI)[a]	p-value	Cases/ Controls	OR (95%CI)[a]	p-value
Thyroid dose before age 15									
<1 mGy	169/207	1[b]		100.133	1 [b]		100/272	1 [b]	
1– 9.9 mGy	138/217	0.8 (0.4; 1.4)		82/133	1.0 (0.4; 2.4)		82/266	1.0 (0.6; 1.8)	
10 – 19.9 mGy	13/10	1.0 (0.3; 3.4)		12/5	3.1 (0.6; 16.5)		12/13	3.0 (1.0; 9.1)	
20 – 35 mGy	4/4	1.5 (0.2; 12.6)		4/2	3.8 (0.3; 63.5)		4/4	4.7 (0.8; 23.5)	
Linear model		0.038 (-0.084 ; 0.16)	0.4		0.21 (-0.23 ; 0.65)	0.02		0.11 (-0.020 ; 0.30)	0.007
Exponential model		0.058 (-0.034 ; 0.14)	0.3		0.18 (0.070 ; 0.29)	0.004		0.094 (0.040; 0.15)	0.004
Quadratic model		0.0032 (-0.0052 ; 0.020)	0.3		0.026 (-0,016 ; 0.068)	0.003		0.0090 (-0.0026 ; 0.026)	0.003

© 2023 de Vathaire F et al. *JAMA Network Open.*

Work at nuclear sites during tests									
No	305/430	1[b]		189/267	1[b]		189/541	1[b]	
Yes	10/8	1.4 (0.5; 4.1)	0.4	9/6	2.0 (0.5; 8.2)	0.3	9/14	2.9 (0.8; 10.3)	0.09

[a]OR: odds ratio, CI: confidence interval. Adjusted for ethnic group, level of education, obesity, family history of thyroid cancer, personal history of radiotherapy, dietary iodine intake, and number of pregnancies for women.

[b]Reference category

.

© 2023 de Vathaire F et al. *JAMA Network Open.*

eTable 10. Papillary Differentiated Thyroid Carcinoma Risk by Lifetime Thyroid Radiation Dose and Nuclear Site Work Status

Exposure	Total population, matched analysis (315 cases and 438 controls)			Excluding unifocal micro carcinomas size and their controls, matched analysis (198 cases / 273 controls)			Excluding unifocal micro carcinomas, unmatched analysis, stratified on study, sex and age (198 cases / 555 controls)		
	Cases/ Controls	OR (95%CI)[a]	p-value	Cases/ Controls	OR (95%CI)[a]	p-value	Cases/ Controls	OR (95%CI)[a]	p-value
Lifetime thyroid dose									
<1 mGy	87/101	1[b]		59/68	1 [b]		59/132	1 [b]	
1– 9.9 mGy	203/306	0.89 (0.50; 1.6)	0.3	119/189	0.68 (0.30; 1.5)	0.07	119/385	1.0 (0.59 ; 1.8)	0.03
10 – 19.9 mGy	20/27	0.99 (0.40; 2.4)		16/14	1.5 (0.44; 4.9)		16/33	1.9 (0.77; 4.6)	
20 – 39 mGy	5/4	1.8 (0.24; 14.4)		4/2	2.5 (0.15; 40.4)		4/5	3.9 (0.76; 20.3)	
Linear model		0.064 (-0.057 ; 0.18)	0.16		0.22 (-0.15 ; 0.61)	0.008		0.075 (-0.027; 0.18)	0.03
Exponential model		0.056 (-0.020 ; 0.13)	0.3		0.15 (0.063 ; 0.214)	0.004		0.071 (0.023; 0.15)	0.02
Quadratic model		0.0021 (-0.0032; 0.0074)	0.2		0.020 (-0,0073; 0.047)	0.003		0.0039 (-0.0015; 0.0093)	0.03

© 2023 de Vathaire F et al. *JAMA Network Open.*

Work at nuclear sites during tests									
No	305/430	1[b]		189/267	1[b]		189/541	1[b]	
Yes	10/8	1.5 (0.49; 4.3)	0.2	9/6	2.1 (0.49 ; 8.7)	0.3	2/13	2.9 (0.84; 10.2)	0.09

[a]OR: odds ratio, CI: confidence interval. Adjusted for ethnic group, level of education, obesity, family history of thyroid cancer, personal history of radiotherapy, dietary iodine intake, and number of pregnancies for women.

[b]Reference category

© 2023 de Vathaire F et al. *JAMA Network Open.*

eTable 11. Radiation Dose Response Modifiers for Dose Received Before Age 15 y

	Total population, matched analysis (395 cases and 555 controls)			Excluding unifocal micro carcinomas and their controls, matched analysis (258 cases / 359 controls)			Excluding unifocal micro carcinomas, unmatched analysis, stratified on study, sex and age (258 cases / 555 controls)		
	Cases/ Controls	OR/mGy (95%CI)[a]	Interaction: p-value[b]	Cases/ Controls	OR/mGy (95%CI)[a]	p-value[b]	Cases/ Controls	OR/mGy (95%CI) [a]	p-value[b]
Study phase									
1	229/373	0.022 (-0.11; 0.16)		147/240	0.17 (-0.31; 0.65)		147/373	0.10 (-0.057; 0.31)	
2	166/182	0.12 (-0.21; 0.45)	0.5	111/119	0.27 (-0.65; 1.18)	>0.5	111/182	0.046 (-0.10; 0.37)	>0.5
Birth period									
Before 1963	176/272	0.069 (-0.18; 0.32)		116/171	0.47 (-0.83; 1.79)		116/272	0.069 (-0.072; 0.25)	
1963 or after	219/283	0.027 (-0.11; 0.16)	>0.5	142/188	0.096 (-0.22; 0.42)	0.2	142/283	0.099 (-0.43; 0.24)	>0.5
Ethnical origin									
Polynesian	219/283	0.073 (-0.10; 0.25)	0.05	142/188	0.18 (-0.18; 0.55)	0.02	142/283	0.12 (-0.047; 0.34)	0.1
Other	176/272	-0.040 (-0.11; 0.25)		116/171	-0.040 (-0.19; 0.23)		116/272	0.011 (-0.081; 0.10)	

© 2023 de Vathaire F et al. *JAMA Network Open.*

Gender									
Female	336/473	0.029 (-0.094; 0.15)	> 0.5	210/293	0.093 (-0.18; 0.26)	>0.5	210/473	0.071 (-0.045; 0.23)	>0.5
Male	59/82	0.14 (-0.43; 0.72)		48/82	0.25 (-0.69; 1.18)		48/82	0.20 (-0.29; 1.81)	
Obesity (BMI>30)									
No	186/355	0.0079 (-0.062; 0.11)	0.2	124/226	0.064 (-0.16; 0.29)	0.5	124/355	0.079 (-0.12; 0.22)	>0.5
Yes	209/200	0.13 (-0.19; 0.41)		134/133	0.17 (-0.26; 0.60)		134/200	0.092 (-0.067; 0.32)	
Number of pregnancies (Women only)									
< 4	145/276	0.12 (-0.18; 0.41)		91/173	0.15 (-0.33; 0.62)		91/276	0.086 (-0.089; 0.26)	
4 or more	191/197	0.043 (-0.15; 0.24)	>0.5	119/120	0.28 (-0.51; 1.08)	0.5	119/197	0.069 (-0.082; 0.22)	>0.5

[a]OR/mGy: Odds Ratio for each mGy, CI: confidence interval. Adjusted for ethnic group, level of education, body surface area, family history of thyroid cancer, personal history of radiotherapy, dietary iodine intake, and number of pregnancies for women.

[b]p-value for interaction.

© 2023 de Vathaire F et al. *JAMA Network Open.*

eFigure. Study Flowchart

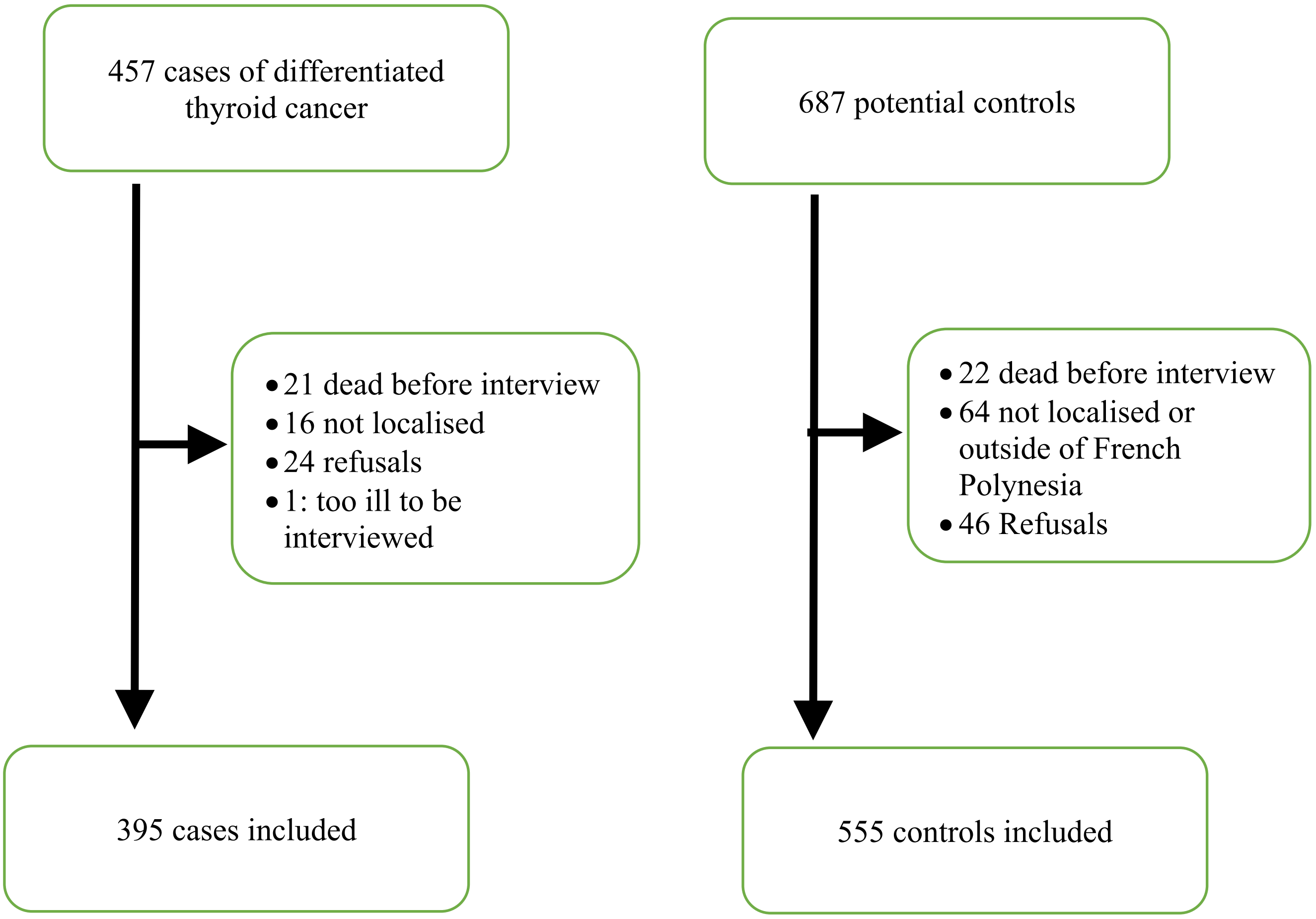

© 2023 de Vathaire F et al. *JAMA Network Open.*
