# Supplementary material for: Assessment of Differentiated Thyroid Carcinomas in French Polynesia After Atmospheric Nuclear Tests Performed by France
Source: JAMA Netw Open. 2023 May 5;6(5):e2311908. doi: 10.1001/jamanetworkopen.2023.11908 (PMC10163383; doi:10.1001/jamanetworkopen.2023.11908)
Supplement: Supplement 2. — Data Sharing Statement [file jamanetwopen-e2311908-s002.pdf]

Data Sharing Statement

de Vathaire. Assessment of Differentiated Thyroid Carcinomas in French Polynesia After Atmospheric Nuclear Tests Performed by France. *JAMA Netw Open*. Published online May 5, 2023. doi:10.1001/jamanetworkopen.2023.11908

Data
Data available: Yes
Data types: Deidentified participant data
How to access data: Deidentified participant data after establishment of a DTA : Florent de Vathaire florent.devathaire@gustaveroussy.fr
When available: With publication

Supporting Documents
Document types: Statistical/analytic code
How to access documents: Florent de Vathaire florent.devathaire@gustaveroussy.fr
When available: With publication

Additional Information
Who can access the data: florent.devathaire@gustaveroussy.fr
Types of analyses: SAS programmes
Mechanisms of data availability: After establishement of a DTA
Any additional restrictions: None

© 2023 de Vathaire F et al. *JAMA Network Open*.
